# Transcriptomic analysis of shell repair and biomineralization in the blue mussel, *Mytilus edulis*

**DOI:** 10.1186/s12864-021-07751-7

**Published:** 2021-06-10

**Authors:** Tejaswi Yarra, Kirti Ramesh, Mark Blaxter, Anne Hüning, Frank Melzner, Melody S. Clark

**Affiliations:** 1 Ashworth Laboratories, University of Edinburgh, Institute of Evolutionary Biology, Charlotte Auerbach Road, EH9 3FL Edinburgh, UK; 2 British Antarctic Survey, Natural Environment Research Council, High Cross, Madingley Road, CB3 0ET Cambridge, UK; 3 GEOMAR Helmholtz Centre for Ocean Research, 24105 Kiel, Germany; 4 Sanger Institute, Wellcome Genome Campus, Hinxton, Cambridgeshire, CB10 1SA Saffron Walden, UK

**Keywords:** Mollusc, Bivalve, Shell matrix proteins, Haemocytes, Calcium

## Abstract

**Background:**

Biomineralization by molluscs involves regulated deposition of calcium carbonate crystals within a protein framework to produce complex biocomposite structures. Effective biomineralization is a key trait for aquaculture, and animal resilience under future climate change. While many enzymes and structural proteins have been identified from the shell and in mantle tissue, understanding biomieralization is impeded by a lack of fundamental knowledge of the genes and pathways involved. In adult bivalves, shells are secreted by the mantle tissue during growth, maintenance and repair, with the repair process, in particular, amenable to experimental dissection at the transcriptomic level in individual animals.

**Results:**

Gene expression dynamics were explored in the adult blue mussel, *Mytilus edulis*, during experimentally induced shell repair, using the two valves of each animal as a matched treatment-control pair. Gene expression was assessed using high-resolution RNA-Seq against a *de novo* assembled database of functionally annotated transcripts. A large number of differentially expressed transcripts were identified in the repair process. Analysis focused on genes encoding proteins and domains identified in shell biology, using a new database of proteins and domains previously implicated in biomineralization in mussels and other molluscs. The genes implicated in repair included many otherwise novel transcripts that encoded proteins with domains found in other shell matrix proteins, as well as genes previously associated with primary shell formation in larvae. Genes with roles in intracellular signalling and maintenance of membrane resting potential were among the loci implicated in the repair process. While haemocytes have been proposed to be actively involved in repair, no evidence was found for this in the *M. edulis* data.

**Conclusions:**

The shell repair experimental model and a newly developed shell protein domain database efficiently identified transcripts involved in *M. edulis* shell production. In particular, the matched pair analysis allowed factoring out of much of the inherent high level of variability between individual mussels. This snapshot of the damage repair process identified a large number of genes putatively involved in biomineralization from initial signalling, through calcium mobilization to shell construction, providing many novel transcripts for future in-depth functional analyses.

## BackgroundK

The molluscan shell is composed of varying proportions of organic components (largely proteins, acidic polysaccharides and chitin) and the calcium carbonate polymorphs: calcite and aragonite. Combined, these give the shell of each mollusc species their unique physical and chemical properties. During shell formation, calcium carbonate is produced from the reaction of calcium ions with bicarbonate ions, and evidence suggests that the proteins (shell matrix proteins or SMPs) determine the mineral polymorph and are involved with the nucleation, growth and termination of the calcium carbonate crystals [1]. SMPs are secreted by the mantle, a layer of tissue between the shell and the rest of the organs it encloses, into the extrapallial fluid, where they are incorporated into the growing edge of the shell along with the calcium carbonate crystals [1]. Hence, the processes of the production of crystal lattices and proteinaceous extracellular matrix are intimately linked in molluscan biomineralization.

SMPs have been identified and characterized in multiple proteomic studies via the extraction of proteins directly from shells. SMPs have been described from several molluscan genera, which have been collated in an in-house SMP database (https://doi.org/10/cz2w [2]). This database contains protein sequences of both putative and known SMPs identified in Uniprot using keyword searches related to molluscan biomineralization (full details in methods). Complementary to these proteomic data, transcriptomic data have been generated from mantle tissue and putative biomineralization loci identified through sequence similarity to already identified SMPs. Transcriptome data have also been deployed to propose source proteins for proteomic mass spectrometry data [3]. The specific roles of SMPs in biomineralization have been explored through functional experimentation. For example, RNA interference mediated knock-down of Pif and PfN23 genes in the mantle disrupted nacre formation in *Pinctada imbricata fucata* [4, 5], while knockdown of the Shematrin gene resulted in disordered foliate structures in *Chlamys farreri* [6]. *In vitro* studies on the effects of SMPs on calcium carbonate crystal formation revealed functional specificity. Pif induced calcium carbonate crystal growth and PfN23 and p10 accelerated crystal growth in *P. imbricata fucata* [4, 5, 7]. In contrast, perlinhibin and perlwapin from *Haliotis laevigata*, prismalin-14 from *P. imbricata fucata* and caspartin from *Pinna nobilis* were found to inhibit crystal growth [4, 8–10]. Although SMPs and mantle transcripts from multiple molluscan species have been identified, there are still many unknowns in the biomineralization process.

Shell matrix proteomics can only identify proteins that are incorporated into the shells and cannot report on enzymic or other upstream processes. Similarly, while mantle transcriptomes have been used to identify putative biomineralization related transcripts, this has largely been based on sequence similarity to previously known SMPs. Importantly, mantle tissue is made up of multiple different cell types with different origins and roles including ectodermal and mesodermal components involved in sensory and muscular functions as well as epidermal and secretory tissue involved in shell formation. This makes it hard to ascertain whether a transcript is involved in biomineralization or in multiple other functions. Species-specific adaptations may also obscure shared biology. For example, it has been proposed that haemocytes, found in the open circulatory system of molluscs, may play an active role during shell formation by carrying amorphous calcium, calcium crystals or SMPs to the site of shell formation [11–13]. However, the involvement of haemocytes in mollusc biomineralization may be species-specific, as they were associated with immune processes in *Crastostrea gigas*, but with ion regulation and calcium transport in *C. virginica* [14]. While *in vivo* and *in vitro* experiments have identified SMPs as integral to shell production, the molecular players in other shell formation processes such as the uptake, mobilisation and storage of calcium and bicarbonate ions, are unclear [15]. Molluscs are proficient at repairing shell damage [16]. Repair of experimentally-induced shell damage has been used in several species to explore the dynamics of the repair process and the genes and proteins involved in biomineralization [17–22]. These previous studies used either pooled individuals or separate controls and treated animals. Therefore part of the aim of this study was to validate the matched pair design using individuals via Illumina RNA-Seq.

The blue mussel *Mytilus edulis* is endemic to European and West Atlantic waters, and is an important species in commercial aquaculture (http://www.fao.org/fishery/species/2688/en). *M. edulis* shells are composed of an outermost organic layer of periostracum, a middle layer consisting of calcite based prismatic structures, and an innermost layer of aragonite based laminar structure called nacre [23]. In this study, samples generated as part of a previously published *M. edulis* shell regeneration experiment [20] were used to measure gene expression changes consequent on damage and repair of adult shells using RNA-Seq transcriptomics. Importantly the experimental model, using within-individual controls enabled identification of differences in gene expression patterns due to the systemic effects of injury and the genetic difference between individuals from those associated with the processes occurring at the wound site. Due to financial constraints and the need for a (relatively) high level of replication (*n* = 5) and to sequence four tissues per animal, this study focused on the time point with the most distinct and homogenous calcification response. A database of genes, proteins and protein domains previously identified as SMPs or associated with SMPs was generated to explore the involvement of these candidates in the shell repair process through time. In addition, comparisons were carried out against *M. edulis* haemocyte expressed sequence tag (EST) datasets to assess the contribution of haemocytes to shell repair and against transcriptome data from *M. edulis* larvae during the synthesis of the first larval shell to validate novel SMPs.

## Results

### Study design

**Fig. 1:**
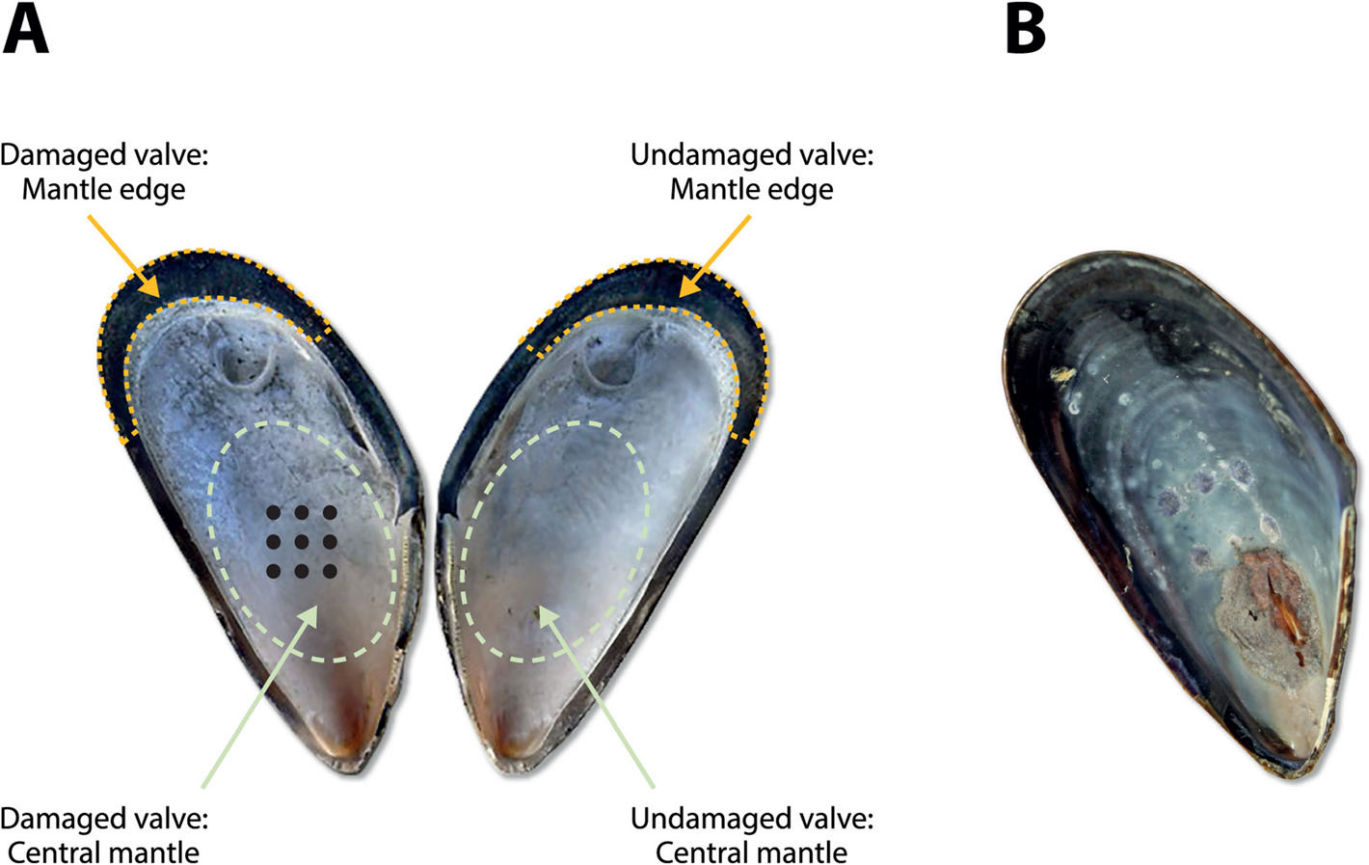
The paired valve design for assessing shell repair in *Mytilus edulis*. **A** Location of drilled holes on the left valve, and the areas of mantle tissue sampled from both valves. **B** Typical extent of healing 29 days after drilling. Picture attributions (**A**) Picture obtained and modified under Creative Commons license (2006) from F. Lamiot, Moule, Miesmuscheln, mussel (anatomia and shell), url: https://commons.wikimedia.org/wiki/File: Moules_Miesmuscheln_mussel3.jpg; (**B**) from Frank Melzner with permission

The tissue samples from five 5 individuals analysed in this study were generated during a longitudinal study of shell repair in adult *M. edulis* [20]. Recent studies have shown that most *Mytilus* populations in Europe are hybrids of *M. edulis*, *M. galloprovincialis* and *M. trossulus*, with varying degrees of admixture [24]. Kiel animals are characterized by a high proportion of *Mytilus edulis* alleles (ca. 80 %) and admixture of *M. trossulus* (ca. 20 %) alleles [25] (Stuckas, Melzner et al. unpublished). A Kiel hybrid transcriptome was assembled and sequenced reads were mapped on this hybrid transcriptome. Since we utilized five replicate animals, we expect that our statistical analyses captured at least the essential transcriptomic signatures related to shell repair. Details of the experimental procedures are given in the original publication, but the salient features are reviewed here. Holes were drilled in the centres of the left valves of a cohort of wild-sampled, live *M. edulis*, above the central mantle zone (Fig. 1A). The right valves were left undamaged. There were no mortalities during the course of the experiment. All individuals successfully initiated repair of the damaged valve (Fig. 1B). By day 29 post-damage, the holes were covered with an outer (water facing) organic layer covering the damaged shell areas, as well as calcitic layers deposited on these, yet no aragonitic layers, as verified by Scanning Electron Microscopy (SEM) and Raman Spectroscopy in the original study [20]. In addition, a PCR-based expression assessment of mantle tissue showed that a key calcite formation gene, nacrein, was highly expressed [20], hence the 29 day time point was appropriate for studying shell repair and deposition of calcite. The mantle edge and central mantle zones of each valve (control and damaged) were collected from five individuals for assessment of differential gene expression at 29 days post-damage, yielding 20 samples in total. Comparison of gene expression in mantle edge and central mantle, within a valve, and between valves within an individual, enabled the isolation of gene expression changes due to the injury-repair processes in the tissue performing the repair (central mantle of the damaged valve) from general processes active in the valve (comparing central versus edge in both damaged and undamaged valves) and systemic processes induced by the repair process (left and right valves in each individual). These within individual data controlled for the expected, large, inter-individual differences in gene expression profiles in *Mytilus* species, which are all outbreeders and highly heterozygous [26, 27].

### Transcriptome assembly, filtering and annotation

Transcriptomic analysis (Illumina RNA-Seq) generated 714 million raw read pairs in total, with 601 million read pairs remaining after adapter trimming and quality and length filtering. Because of the high genetic variability between *M. edulis* individuals and haplotypes, and thus poor mapping of reads from individuals in this study to previously generated transcriptomic and genomics data, a *de novo* transcriptome was assembled to act as reference. The pooled, cleaned read set was down-sampled to 31 million read pairs by *in silico* normalization. These were assembled using the Trinity pipeline into 560,776 putative genes with 874,699 transcript fragments (likely isoforms). Filtering of the assembly to eliminate expression noise (including putative genes only if they had more than 1 mapped read per million mapped reads in at least 10 libraries) yielded 30,822 putative genes, with 158,880 transcript fragments (Table 1). These data are similar in magnitude to a recently produced *M. edulis* transcriptome, which also sourced animals from the Baltic [28]. Reads were aligned from each sample to this filtered reference and gene expression was assessed by summing the counts of mapped read pairs per putative gene.

**Table 1:** Mantle transcriptome assembly metrics

**Main assembly**	
Trinity genes	560,776
Trinity transcripts	874,699
**Filtered assembly (> 1 CPM in ≥ 10 libraries)**	
Trinity genes	30,822
Trinity transcripts	158,889
Protein sequences (ORF ≥ 100 amino acids)	81,456
**Filtered assembly features**	
% GC	33.54
N50 (bp)	1,602
Minimum length (bp)	201
Maximum length (bp)	26,467
Total assembled bases (Mbp)	181

### Differential gene expression

**Fig. 2:**
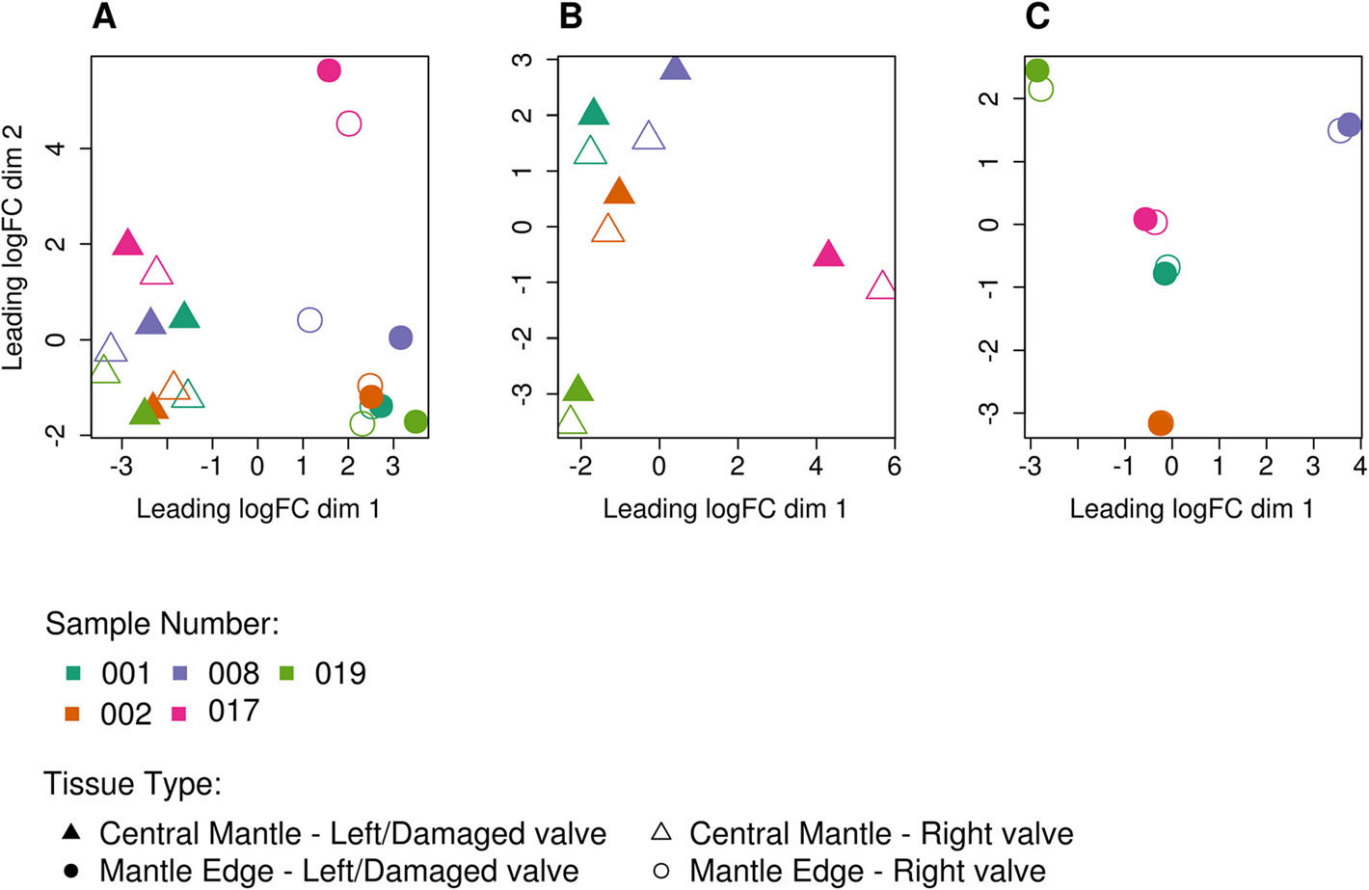
Multidimensional scaling identifies significant contributions of individual variation to gene expression differences in shell repair in *Mytilus edulis****.*** MDS plots of expression counts for the filtered set of putative genes in (**A**) All libraries: Central mantle – left/damaged valve; Central mantle – right undamaged (control) valve: Mantle edge – left/damaged valve; Mantle edge – right undamaged (control) valve, **B** Central mantle libraries only (**C**) Mantle edge libraries only

**Fig. 3:**
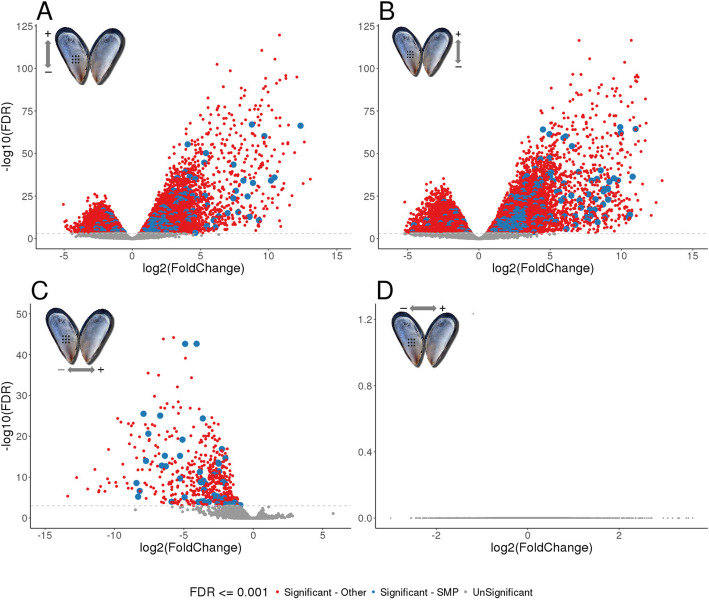
Differential gene expression in *Mytilus edulis* mantle tissues during shell repair. Volcano plots detailing differential gene expression between the four mantle tissue libraries. Inset mussel pictures show comparisons detailed in each plot. **A** Damaged valve: mantle edge versus central mantle, **B** Control valve: mantle edge versus central mantle, **C** Damaged central mantle versus control central mantle, **D** Damaged mantle edge versus damaged central mantle. Dashed lines indicate the FDR value of 0.001. Note: The axis scales are not the same across all plots

Multidimensional scaling (MDS) plots of the digital expression levels showed separation between mantle edge and central mantle tissues in dimension 1, with dimension 2 roughly corresponding to different individuals (Fig. 2A). There was a significant difference in expression levels in the central mantle both between damaged and undamaged valves and between individuals (Fig. 2B). Although the expression levels of mantle edge libraries also showed separation between different individuals, there was no significant difference between the damaged and undamaged valves (Fig. 2C). Four pairwise comparisons were made for differential gene expression between the tissues and valves (Table 2; Fig. 3). In both the damaged and undamaged valves, many putative genes were found to be differentially expressed between the mantle edge and the central mantle (Fig. 3A,B). When the mantle of the damaged and undamaged (control) valves were compared, 653 transcripts were highly expressed in the central mantle of the damaged valve during shell repair, with 54 of these transcripts having sequence similarity with SMPs (Fig. 3C, Table 2). No putative genes were identified as differentially expressed between the mantle edge tissues of damaged and control valves (Fig. 3D).

**Table 2:** Number of differentially expressed contigs between mantle tissue sections and annotation levels

Comparison	Differential expression		Annotation^**b**^		
	Tissue in which genes are more highly expressed	FDR^**a**^ <=0.001	SwissProt	Trembl	SMP database
Undamaged valve: mantle edge vs. central mantle	Mantle edge	8,955	2,293	4,001	220
	Central mantle	7,221	3,128	3,780	23
Damaged valve: mantle edge vs. central mantle	Mantle edge	7,340	2,039	3,484	155
	Central mantle	6,229	2,614	3,144	28
Central mantle: Damaged vs. undamaged valve	Damaged valve	653	131	236	54
	Undamaged valve	0	0	0	0
Mantle edge: Damaged vs. undamaged valve	Damaged valve	0	0	0	0
	Undamaged valve	0	0	0	0

^a^*FDR* False Discovery Rate

^b^number of putative genes with annotation derived through sequence similarity searches of the stated databases

### Annotation of transcripts associated with damage-repair

**Fig. 4:**
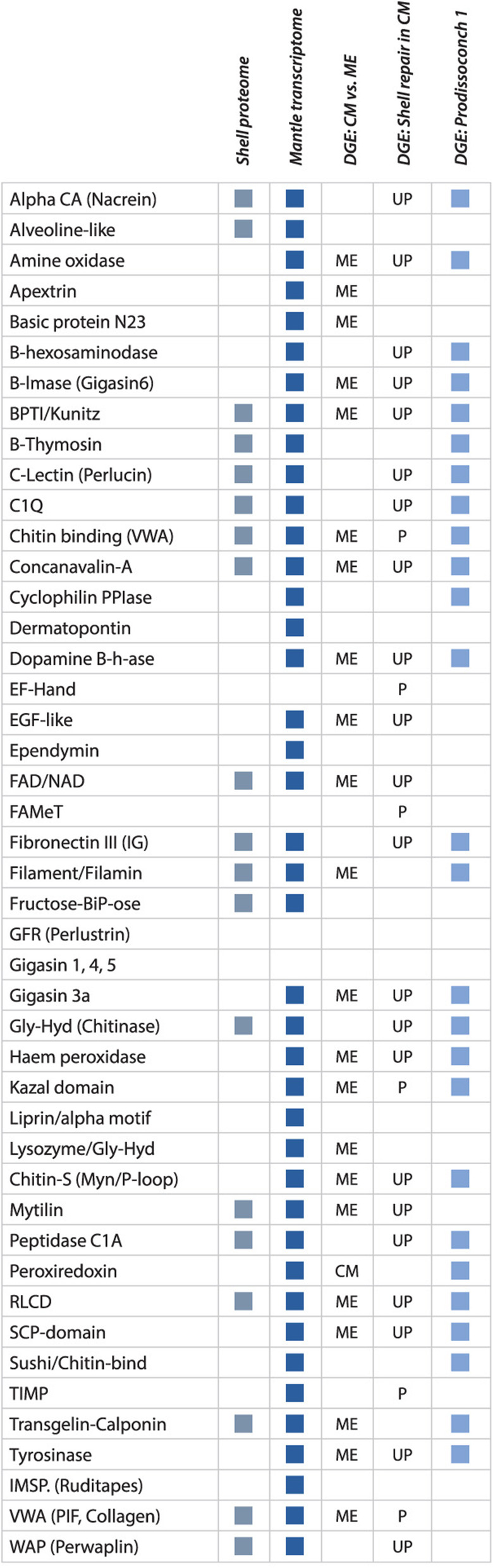
Shell matrix protein homologues identified in *Mytilus edulis* shell proteomes, transcriptomes, and differential gene expression. For each identified protein or protein domain the columns indicate: Shell proteome: Previously identified shell proteome sequences; Mantle transcriptome: Transcripts previously identified in mantle transcriptome studies; DGE: CM vs. ME: Differential gene expression (DGE) identified in the central mantle (CM) versus the mantle edge (ME); DGE: shell repair in CM: Trajectory of DGE in the central mantle (UP = up-regulation; P = putative shell proteins with no strong sequence similarity to, but with similar functional domains to known SMPs); DGE: Prodissoconch I: Genes differentially expressed in the prodissoconch I in transcriptomic analysis of development. The haemocyte dataset has not been included, as only one domain (C1Q) in common was identified

**Fig. 5:**
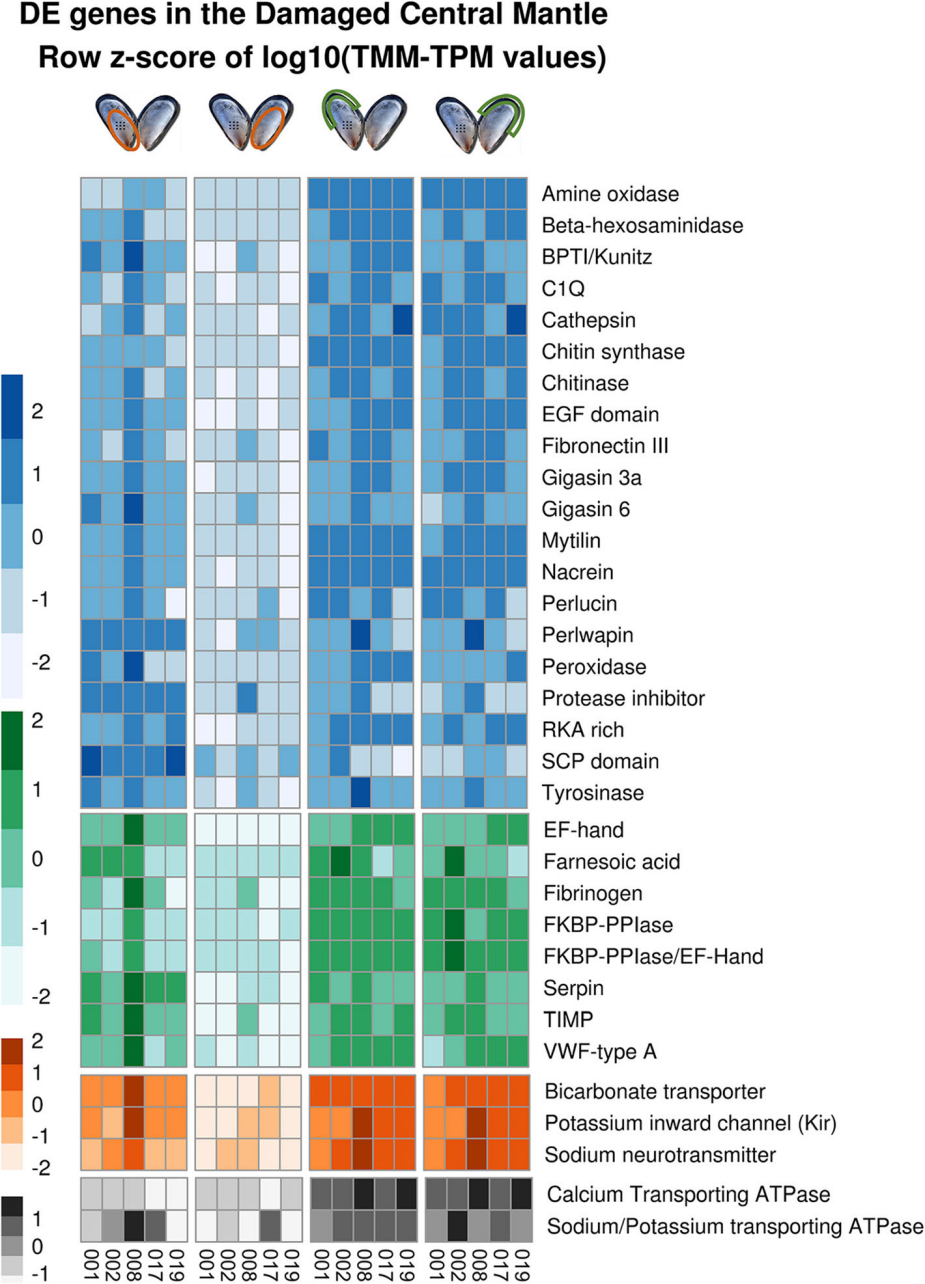
Expression of selected sets of differentially expressed genes in central mantle during shell repair in *Mytilus edulis*. For each differentially expressed gene set (rows) four sets of five columns show the fold expression change in each of the five individuals (001–005). The sets of columns from left to right are: Damaged central mantle, Control central mantle, Damaged mantle edge, and Control mantle edge. The differentially expressed gene sets are grouped and colour coded: Blue: DE genes with sequence similarity to SMPs, ordered by SMP name; Green: DE genes with domains found in SMPs, but no sequence similarity to known SMPs, ordered by domain name; Orange: DE genes containing transmembrane domains; Grey: Non-DE genes with sequence similarity to ATPases of interest

Further in-depth analysis was restricted to the 653 putative genes associated with the comparison of damaged and control central mantle tissues (Fig. 3C, Table 2), as these were most likely to be involved in damage-repair. All 653 genes were upregulated in the damaged valve undergoing repair. Gene ontology analysis of these 653 genes showed enrichment, compared to the total putative gene translation dataset of several molecular processes associated with protease inhibition (including serine-type endopeptidase inhibition), chitin-binding and metalloendopep tidase activity (Table 3). Sequence similarity searches identified specific transmembrane transporters, proteases and protease inhibitors, signalling molecules and tyrosinases in this gene set (Figs. 4 and 5). Just over 8 % (54 of 653) of these putative genes had sequence similarity with known SMPs or domains associated with SMPs (Fig. 4). In addition to identification of homologues of previously described SMPs, we identified a number of putative genes that had no strong sequence similarity to known SMPs but contained SMP-associated domains such as VWA (chitin-binding), EF-hand, FAMeT, Kazal, and TIMP (Fig. 4).

**Table 3:** Enriched Molecular Function GO terms in differentially expressed genes in damaged and undamaged valves

**Undamaged mantle edge**	**Damaged left valve**
G-protein coupled receptor activity Calcium ion binding Protein binding Sequence-specific DNA binding Ion channel activity	Peptidase inhibitor activity Chitin-binding Serine-type endopeptidase inhibitor Metalloendopeptidase inhibitor
**Undamaged central mantle**	**Undamaged right valve**
Nucleic acid binding Nucleotide binding ATP binding Metal ion binding	None

The initial stages of embryonic shell formation in *M. edulis* are characterised by the deposition of aragonite, while the adult shell has both calcite and aragonite microstructures. However, analyses in other species such as the gastropod *Lymnaea stagnalis* and the oysters *P. imbricata fucata* and *Crassostrea gigas* have revealed similarities in gene expression repertoires between adult and larval shells [29, 30]. Many of the differentially expressed genes with SMP annotations identified in this study were also differentially expressed in the transcriptomics dataset from the prodissoconch I stage of *M. edulis* developing larvae (Fig. 4) [31] (Fig. 4). Furthermore, to identify whether haemocytes could be involved in shell repair processes, 2,194 sequences from a *Mytilus* haemocyte EST dataset were extracted from MytiBase [32] and compared with the current dataset. Only one sequence with one of the SMP-associated domains (C1Q) was identified in both datasets. Thus evidence for haemocyte involved in damage repair is limited in *M. edulis.* Interestingly, transcripts highly expressed in the central mantle of the damaged valve during shell repair were also present in the mantle edge transcriptomes and with similar expression levels, suggesting a general similarity in function (Fig. 5).

## Discussion

Biomineralization is a complex process, and subject to developmental and environmental control. Using a carefully internally-controlled gene expression analysis, this study identified a large number of putative genes that may be involved in coordinating and carrying out shell repair in *M. edulis*, an important ecosystem and aquaculture species. Importantly the experimental design controlled for the known high genetic variation in *M. edulis* [2, 26, 27] by exploiting the bivalve condition and using a matched pair analysis, whereby the control and treated (damaged) samples were taken from the same individual (Fig. 1) [20]. The sampling regime minimised individual effects (both genetic and environmental) on signal discovery, as confirmed by the MDS plots, in which the variability between individuals was much larger than the difference between experimental and control groups (Fig. 2A-C) [2]. To optimise the detection of genes of interest, a modified damage-repair protocol was employed [6, 17–22]. A series of holes were drilled in the central region of the *M. edulis* shell to induce repair processes. Morphological assessment showed that by day 29 the central mantle had produced effective repair of the shell, including the deposition of calcite [20]. Normally it is the mantle edge tissue that is integral to active shell growth and the secretion and deposition of calcium carbonate. In contrast, in normal conditions the role of the central mantle is to maintain the shell (as shown by the differences in expression profiles in Fig. 5 between undamaged and damaged mantle tissue). The two areas of mantle tissue also have very different anatomies, with the central mantle being a thin layer of epithelial tissue encompassing the animal and the mantle edge comprising a more complex folded structure, typically comprising three folds and periostracum in most bivalves [33, 34]. The large differences in the numbers of differentially expressed genes between the mantle edge and the central mantle (Table 2; Fig. 3) and associated GO enrichments (Table 3) highlighted their distinct physiological roles. However, the mantle is a multifunctional organ and it is unlikely to be possible to identify biomineralization-specific genes solely on differential expression between the mantle edge and central mantle. Previous studies have examined mantle edge responses to damage and modulation during growth, and thus risk confounding normal growth and repair.

The current experimental design was based on the hypothesis that inducing shell repair in the central mantle would specifically invoke expression of genes normally expressed by the mantle edge associated with shell production. By assessing the response of central mantle tissue to damage, switching from a low level of maintenance to active repair and reconstruction, it was possible to identify signals specific to the biomineralization process. Similarly, as wounding may induce whole-organism stress and immune processes, the undamaged valve was used as a within-individual control to remove systemic gene expression responses (Fig. 5) [2]. All sampled *M. edulis* were healthy and active at the time of sampling, suggesting that the experimental damage had not resulted in major systemic infection or necrosis. Multidimensional scaling analysis identified inter-individual variation as a major component describing expression level variation, and inter-individual variation was much larger than the difference between experimental and control valves (Fig. 2A-C) [2]. This approach should also be effective in analysis of other traits in this and other species of bivalve.

In mollusc damage-repair experiments, the level of response in the mantle tissue can depend heavily on where the damage was caused relative to where the mantle tissue was sampled [22]. In the current experiment gene expression of the mantle edge in the damaged valve was not affected during repair (Fig. 3D) suggesting that at this late stage of the repair process, gene expression effects were localized to tissue at the area of damage. This does not mean that mantle edge tissue did not respond to damage or was not involved in repair, but that the repair occurring in the this region of the mantle did not result in changes in gene expression over the normal biomineralization programmes active in this tissue. Many genes that were highly expressed in the central mantle of the damaged valve during shell repair also had high expression in the mantle edge (Fig. 5). Thus, the functions of the central mantle can transition to resemble those of the mantle edge during shell healing, in keeping with observations of altered mantle tissue ultrastructure during shell repair in bivalves [35, 36]. As thickening and repair of central shell parts occur in adult *M. edulis*, for example in response to high predator densities, shell boring polychaetes such as *Polydora* species, or in response to specific local habitat conditions [37–39], this phenotypic plasticity is of adaptive significance.

The hypothesis of critical involvement of haemocytes in repair-associated biomineralization [11–14] was not supported in *M. edulis*. Cross comparison of the genes highly expressed in the central mantle during shell repair with an EST dataset generated from *Mytilus* haemocytes identified very few shared genes, highlighting their different functional repertoires. Molluscs have an open circulatory system, where the haemocytes are not confined to the haemolymph and are free to move into surrounding tissues and mantle cavity [40]. At a general functional level, only three domains (C1Q, tumour necrosis factor-like (TNF) and FN3) were found in proteins expressed in both haemocytes and adult mollusc SMPs. These domains are associated with proteins involved in the mollusc, and other non-vertebrate, immune responses [41–43]. Genes encoding these domains were highly expressed in the mantle edge compared to the central mantle in the control valve, suggesting a higher level of haemocyte activity in the mantle edge compared to the central mantle (Fig. 4). This is consistent with the positions of these tissues in the animal and their different functions. The mantle edge faces the external environment and therefore would be expected to require increased levels of immune defence compared with internal tissues. The identification of immune-related domains within shell proteomes has led to the suggestion that shells are not only structurally protective, but may also play a role in biochemical defence [44].

Previous analyses of diverse mollusc gene sets has shown that some genes involved in biomineralization can be highly divergent between species [45], and genes involved in shell production can be members of lineage-restricted protein families or unique adaptations of conserved genes through the acquisition of new domains and domain shuffling [46]. In this study a dataset of experimentally determined SMPs, and the protein functional domains within those proteins was produced and is openly available at https://doi.org/10/cz2w. Screening the *M. edulis* mantle transcriptomes for sequences homologous to these genes or containing these domains provided a primary list of several hundred candidate SMPs, and this candidate set was further refined through differential expression analysis. In the 653 putative genes (2 % of all putative genes) whose expression was specifically modulated following to damage to the shell, half (325) had significant similarity to previously determined protein sequences, including SMPs. Among the 328 putative genes that had no significant similarity to other proteins, an additional 10 % were detected with similarity to protein domains previously associated with shell formation. Many of these unknowns encoded predicted proteins with secretory leader peptides (39 sequences), coiled domains (16 sequences) and natively disordered regions (91 sequences, 14 % of all differentially expressed genes). Natively disordered regions are characteristic of repetitive low complexity domain proteins (RLCDs), which are often present in shell proteomes and transcriptomes in high numbers as a result of species-specific expansions [47–49]. The identification of 91 such domains in this dataset (almost 14 % of damage-repair differentially expressed sequences) indicated that similar expansions of RLCD families have also occurred in *Mytilus.*

Many of the repair-upregulated genes had functional annotations previously indicated as important in biomineralization, but this study identified further annotations that extend this model. Many repair-upregulated genes had annotations associated with carbohydrate-binding: C-type lectin, beta-hexosaminidase, glycosyl hydrolase, chitinase and chitin-binding. Of particular interest was the identification of chitin-binding, which was also one of the GO terms enriched in the central mantle during repair (Table 3). Support for a role of chitin in the shell comes from experiments examining the effects of chitinase inhibitors on adults and larvae of the freshwater gastropod, *Lymnaea stagnalis* and the mussel *Mytilus galloprovincialis.* Treatment resulted in thinner shells and malformations [50, 51]. Chitin-binding domains are also found in the SMPs Pif97 and blue mussel shell protein (BMSP). These two proteins also have conserved von Willebrand factor A domain (vWA) domains [52], and vWA domains were found in several additional *M. edulis* repair-upregulated genes. Other protein-protein interaction domains found in SMPs such as epidermal growth factor (EGF), fibronectin type III (FN3) and whey acidic protein repeats (WAP) were also found in otherwise novel repair-upregulated genes (Fig. 4). VWF, along with FN3 is involved in cell adhesion and wound healing [53, 54]. Epidermal growth factor (EGF) domains are found in gigasin-2 and other EGF-like proteins and is a common domain in secreted or membrane bound proteins [23, 55]. Tyrosinase proteins were also up-regulated during repair. These proteins are critically involved in the formation of the periostracum, the initial organic layer integral to calcium carbonate deposition [56]. It was perhaps, not surprising to identify three domains (chitin-binding, vWA and tyrosinase) along with carbonic anhydrase (another domain expressed in this damage-repair study), in the up-regulated gene set. These are all members of a proposed universal molluscan biomineralization tool kit, a core set of protein domains shared between all bivalves irrespective of calcium carbonate polymorph and microstructure [44]. Other SMPs, highly expressed during shell repair included the SCP domain, first identified in *Lottia* and Gigasin 3a from *Crassostrea* [23, 57]. These two domains were also identified in the *Mytilus* prodissoconch I transcriptome [31].

GO analysis identified other processes active during *M. edulis* shell repair and deposition. Peptidase inhibitor activity, serine-type endopeptidase inhibition and metalloendopep tidase activity were enriched in the central mantle (Table 3) [58]. These GO terms are associated with known SMPs such as perlwapin, BPTI/kunitz, alpha-2-macroglobulin, kazal and WAP-type ‘four-disulfide core’ domains, tissue inhibitor of metalloproteinase (TIMP) and serine protease inhibitors (Serpins). Furthermore, these domains are all generally found in proteins with proteinase inhibitor activity. Proteases and protease inhibitors were shown to be directly involved in the nucleation and, or, growth and termination of crystal calcification, respectively. For example, serine proteases promote mineralization in vertebrates and bacteria, with serine protease inhibitors controlling this mineralization [59, 60]. In addition, metallopeptidases have been shown to assist in enamel calcification in humans [61] and perlwapin inhibits growth of nacre crystals [9].

Genes with potential enzymatic functions found to be upregulated during repair included several known biomineralization enzymes such as carbonic anhydrase and tyrosinase, but some repair-upregulated genes were annotated with functions not previously strongly associated with biomineralization (Fig. 4). The rediscovery of known biomineralization genes supported the assertion that the novel genes are very likely also biomineralisation toolkit loci. Genes predicted to encode proteins with a farnesoic acid O-methyltransferase (FaMeT) domain were upregulated in the repairing tissue. FaMeT catalyzes the formation of methyl farnesoate from farnesoic acid. Methyl farnesoate is an important hormone protein in crustaceans, with possible roles in moulting [62]. The FaMeT domain was previously identified in SMPs from the gastropod *Haliotis* [47] and these findings in a bivalve suggest that FaMeT involvement in biomineralisation process may be more widespread in molluscs. An amine oxidase (AO) was upregulated in the repairing tissue. AO was implicated in shell production during larval growth of the pearl oyster *P. fucada* [63] and this finding in *M. edulis* suggests that AO involvement in biomineralisation may be more general.

To orchestrate the expression of structural and enzymatic proteins for shell repair, the mollusc must modulate pathways of intra- and inter-cellular signalling and ion balance, but these will not necessarily be evident in SMP analyses. In this study, a number of genes were identified with annotations associated with intra- and inter-cellular signalling in the repair-upregulated set, including a rhodopsin-like G-protein coupled receptor (GPCR), frizzled-like domain and serine-threonine and tyrosine kinases. Whilst GPCRs have previously been identified in shell transcriptomes and have a known role in vertebrate calcium metabolism [64], specific involvement of rhodopsin-like GPCRs and frizzled domains have not previously been established in biomineralization experiments. Serine-threonine kinases are important in biomineralization of teeth and bones in vertebrates [65] and tyrosine kinases are important in phosphorylation of proteins secreted to the extracellular space [66]. Hence, there is the suggestion from vertebrate studies that their roles may be, at least partially, conserved in invertebrates.

Mantle tissue is responsible for calcium turnover and calcium deposition in the shell of molluscs [67] and this process requires active ion transport against environmental gradients and between cells. In the oyster *C. gigas* treatment of mantle tissue *in vitro* with the calcium channel inhibitor verapamil identified some of the entry into the outer mantle through L-type and T-type voltage-gated calcium channels located in the basolateral membrane [68, 69]. However, as verapamil only reduced calcium transport by 20 %, other calcium transporting proteins are likely to be involved. Secretion of Ca^2+^ ions into the extrapallial space across the apical membrane was demonstrated via calcium ATPases and Na^+^/Ca^2+^-exchangers [68, 69]. Previous mantle transcriptome studies have shown that Na^+^/K^+^ ATPase and bicarbonate transporters are upregulated during shell production [29, 58]. In the experiment reported here, calcium transporting ATPases and sodium-potassium transporting ATPases were highly expressed in both repairing and control mantle edge tissue, but were not significantly overexpressed in repairing central mantle (Fig. 5). Solute carrier 4 bicarbonate transporters (SLC4 family members), sodium neurotransmitter symporters (SNSs) and inwardly rectifying potassium channels (Kirs) were identified in the repair-upregulated gene set. SNS belong to the solute carrier 6 gene family and are found in the plasma membrane of neuronal or neuroglial cells, where they are involved in the removal of neurotransmitters from the extracellular space, deriving energy for the uptake from the co-transport of Na^+^ ions along the concentration gradient [70]. Kirs selectively mediate movement of K^+^ ions from the extracellular space into the cell, against a K^+^ gradient [71]. Kir channels are expressed in epithelial cells during osteoblastogenesis in humans [72], and in the freshwater ramshorn snail *Planobarius corneus* neuronal Kir channels maintain the resting potential of membrane in steady state and perturbation conditions [73]. Upregulation of expression of SNS and Kir loci suggests active neural involvement in repair, possibly maintaining membrane potential in the face of the considerable movement of charged ions required during shell repair.

## Conclusions

Using a shell damage-repair model and a newly developed SMP and SMP-associated domain database, novel loci were identified with likely roles in biomineralization in the important bivalve *M. edulis.* A matched pair analysis to reduce the inherent high level of variability between individuals greatly facilitated the identification of genes that were differentially expressed during shell repair, identifying a large number of genes putatively involved in biomineralization, including several previously identified shell matrix proteins. Importantly this study extended the analysis of biomineralisation from the enzymatic and structural players in the shell matrix deposition process itself to loci likely to be involved in associated ion balance and signalling pathways. Our study provides new candidates for functional genomic and reverse-genetic analysis of mollusc biomineralization.

## Methods

### Experimental design

The shell damage-repair experiment is described in detail in a previous study [20] and comprised a total of 45 blue mussels (*Mytilus edulis*) sampled under different experimental conditions. In summary, *M. edulis* were acquired from the Kiel Fjord, Germany (54°19.8’N,10°9.0’E) between April 7–12 2011. Nine holes of 1mm in diameter were drilled (using drill N62/E, Proxxon, Germany) into the central area of the left valve while ensuring the animal soft tissue inside the shell was not harmed. The drilled mussels were suspended in Kiel Fjord in net cages (mesh diameter: 15mm) in 2 m depth, thus ensuring sufficient supply with planktonic food. Temperatures close to the cages rose from ca. 5–12 °C during the regeneration period (April – May 2011), pH (> 8.1–8.3), but salinity (13–16) fluctuated randomly (see Figure S1 in [20]). Mantle tissue was sampled 29 days after drilling. Mantle tissue from the edge and central areas of both valves was collected separately for RNA extraction and sequencing (Fig. 1A).

### RNA extraction and sequencing

Total RNA from the mantle tissues (*n* = 5 individuals, 4 tissue sections each: damaged valve mantle edge and central mantle and control valve mantle edge and central mantle) was extracted according to [74]. Complementary DNA (cDNA) was synthesized using the SMART cDNA synthesis kit (Clontech Laboratories, Mountain View, USA) with quality control performed using the Experion Automated Electrophoresis System (Bio-Rad,Hercules, USA) and the Nanodrop spectrophotometer (Thermo Scientific, Waltham, USA) for RNA as well as using the Bioanalyzer 2100 (Agilent Technologies, Santa Clara, USA) for cDNA. Non-stranded libraries were prepared using the TruSeq RNA Library Prep Kit (including polyA selection; Illumina, San Diego, USA). The indexed libraries from each sample were pooled at equimolar concentrations and sequenced on three HiSeq2000 lanes (Illumina, USA) following a 2 × 125 bp paired-end protocol at the University of Kiel Sequencing Facility at the Institute of Clinical Molecular Biology (IKMB) [26].

### Bioinformatics analysis

All bioinformatic analyses were carried out using default software parameters unless otherwise specified. Adapters were trimmed from raw reads using Trimmomatic v.0.33 [75] and quality- and length-based trimming was performed using Fastq-mcf v.1.04.636 [76], setting the Phred quality score to 30 and minimum read length to 80 b. Cleaned reads were normalized *in silico* with a coverage value of 30 (-max cov) and assembled using Trinity v.2.2.0 [77] with the max kmer cut-off value set to 2. Non-normalized cleaned reads were then aligned to the de novo transcript assembly with Bowtie v.1.1.1 [78] and expression level estimation of putative genes was calculated using RSEM (RNA-Seq by Expectation-Maximization) v.1.2.20) [79]. Raw counts, and counts normalized using trimmed mean of maximum-values (TMM) and transcripts per million (TPM) were generated [80]. Differential gene expression analysis was performed using edgeR v.3.12.1 [81]. Raw counts were used for differential expression assessment, as edgeR performs its own sample normalization. Putative genes from the Trinity assembly that had fewer than 1 counts per million (CPM) read mapping values in at least 10 libraries were removed prior to analysis, as very low count values interfere with statistical approximations and exaggerate fold-change calculations [81]. Differential gene expression was assessed using an additive model to account for the paired experimental design (individual and tissue), and only results with an FDR of at least 0.001 were considered.

Contigs based on CPM-filtered putative genes were translated into putative protein sequences using Transdecoder in the Trinity pipeline. Translations shorter than 100 amino acids were discarded. The transcripts and protein sequences were annotated using multiple tools, including sequence similarity searches using BLAST (blastx or tblastx) v.2.2.30 [82] and domain searches using Interproscan v.5.25-64.0 [83]. BLAST searches were performed with an E-value cut off of less than 1e-10 against both protein (SwissProt, Trembl, our in-house SMP database (https://doi.org/10/cz2w)) and nucleotide (haemocyte expressed sequenced tags; [30], *Mytilus* larval transcriptome [29]) databases. BLAST matches postfiltered to excclude matches that covered less than 40 % of the database entry. Domains and motifs were identified in the translated protein sequences and gene ontology (GO) terms assigned using Interproscan and Interpro [83, 84]. Enrichment of GO terms was assessed using the Trinity Trinotate and GOSeq, with a FDR value of 0.001. TMM normalized TPM count values were used to generate heatmaps.

### Shell Matrix Proteins database

An in-house molluscan Shell Matrix Proteins (SMP) database was developed to aid annotation (https://doi.org/10/cz2w [2]). SMPs of multiple species were downloaded from Uniprot (http://www.uniprot.org/) using keywords related to molluscan biomineralization (molluscs, shell, bivalve, aragonite, calcite, prismatic, foliated, mantle, mantle edge, central mantle, pallial mantle). The SMP dataset was manually curated by reviewing the publication related to each protein entry, and only entries that were validated to be present in molluscan shell matrices were retained. Sequences that were initially selected because they were only mantle-specific were not included. The SMP database contains 327 SMPs from molluscan genera. Domains found in the proteins in the SMP database were annotated Interproscan v.5.25-64.0 [83]. SMP database entries were grouped by functional domain, to reconcile differing naming conventions in previous studies.

## Data Availability

The sequence dataset supporting the conclusions of this article is available in NCBI SRA (Short Read Archive) (https://www.ncbi.nlm.nih.gov/sra) under accession number SRP108359. The SMP database is publicly available from the NERC Polar Data Centre repository (https://www.bas.ac.uk/data/uk-pdc/): GB/NERC/BAS/PDC/01132 with DOI: https://doi.org/10/cz2w.

## Abbreviations

AO: Amine oxidase
BMSP: Blue mussel shell protein
cDNA: Complementary DNA
CPM: Counts per million
EGF: Epidermal growth factor
EST: Expressed sequence tag
FDR: False discovery rate
FN3: Fibronectin type III domain
GO: Gene ontology
Kir: Inwardly rectifying potassium channel
MDS: Multidimensional scale
RLCD: Repetitive low complexity domain protein
SEM: Scanning electron microscopy
SMP: Shell matrix protein
SNS: Sodium neurotransmitter symporter
TMM: Trimmed mean of maximum-values
vWA: Von Willebrand factor A domain
WAP: Whey acidic protein
